# Injectable “Homing‐Like” Bioactive Short‐Fibers for Endometrial Repair and Efficient Live Births

**DOI:** 10.1002/advs.202306507

**Published:** 2024-03-19

**Authors:** Yumeng Cao, Jia Qi, Juan Wang, Liang Chen, Yuan Wang, Yijing Long, Boyu Li, Junliang Lai, Yejie Yao, Yiwen Meng, Xiaohua Yu, Xiao‐Dong Chen, Lai Guan Ng, Xinyu Li, Yao Lu, Xiaoyue Cheng, Wenguo Cui, Yun Sun

**Affiliations:** ^1^ Department of Reproductive Medicine, Ren Ji Hospital Shanghai Jiao Tong University School of Medicine Shanghai 200135 P. R. China; ^2^ Shanghai Key Laboratory for Assisted Reproduction and Reproductive Genetics Shanghai 200135 P. R. China; ^3^ Department of Orthopaedics Shanghai Key Laboratory for Prevention and Treatment of Bone and Joint Diseases Shanghai Institute of Traumatology and Orthopaedics Ruijin Hospital Shanghai Jiao Tong University School of Medicine Shanghai 200025 P. R. China; ^4^ Hangzhou Phil Stone Biotech Co., Ltd. Hangzhou Zhejiang 311215 P. R. China; ^5^ Department of Comprehensive Dentistry University of Texas Health Science Center at San Antonio San Antonio TX 78229 USA; ^6^ Research Service South Texas Veterans Health Care System Audie Murphy VA Medical Center San Antonio TX 78229 USA; ^7^ Shanghai Immune Therapy Institute Shanghai Jiao Tong University School of Medicine affiliated Renji Hospital Shanghai 200127 P. R. China

**Keywords:** ECM short‐fibers, endometrial repair, fertility enhancement, intrauterine injection

## Abstract

The prevalence of infertility caused by endometrial defects is steadily increasing, posing a significant challenge to women's reproductive health. In this study, injectable “homing‐like” bioactive decellularized extracellular matrix short‐fibers (DEFs) of porcine skin origin are innovatively designed for endometrial and fertility restoration. The DEFs can effectively bind to endometrial cells through noncovalent dipole interactions and release bioactive growth factors in situ. In vitro, the DEFs effectively attracted endometrial cells through the “homing‐like” effect, enabling cell adhesion, spreading, and proliferation on their surface. Furthermore, the DEFs effectively facilitated the proliferation and angiogenesis of human primary endometrial stromal cells (HESCs) and human umbilical vein endothelial cells (HUVECs), and inhibited fibrosis of pretreated HESCs. In vivo, the DEFs significantly accelerated endometrial restoration, angiogenesis, and receptivity. Notably, the deposition of endometrial collagen decreased from 41.19 ± 2.16% to 14.15 ± 1.70% with DEFs treatment. Most importantly, in endometrium‐injured rats, the use of DEFs increased the live birth rate from 30% to an impressive 90%, and the number and development of live births close to normal rats. The injectable “homing‐like” bioactive DEFs system can achieve efficient live births and intrauterine injection of DEFs provides a new promising clinical strategy for endometrial factor infertility.

## Graphical abstract

The injectable “homing‐like” bioactive decellularized extracellular matrix short‐fibers (DEFs) derived from porcine skin are innovatively developed for the purpose of endometrial and fertility restoration. Through the release of bioactive factors, the DEFs effectively promote endometrial repair, angiogenesis, and reduce collagen deposition. This mechanism ultimately facilitates the restoration of fertility, leading to the achievement of high‐efficiency live births.

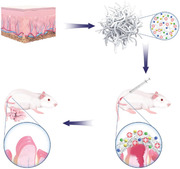

## 1 Introduction

Infertility has become a non‐negligible public health issue, and been estimated to affect ≈15% of the global population.^[^ ^1^, ^2^ ^]^ An embryo‐receptive endometrium is one of the key determinants of a successful pregnancy.^[^ ^3^, ^4^, ^5^ ^]^ Various factors, such as infection and intrauterine surgery, can lead to damage to the basal layer of the endometrium, which hinders functional endometrial repair and results in the pathological development of intrauterine adhesion (IUA). Common manifestations of IUA include thin endometrium (TE), fibrosis, reduced endometrial receptivity,^[^ ^6^, ^7^, ^8^, ^9^, ^10^ ^]^ and consequently, a low embryo implantation rate and live birth rate.^[^ ^11^ ^]^ Current therapeutic approaches for the treatment of endometrial damage consist of hysteroscopic intrauterine adhesiolysis,^[^ ^12^ ^]^ implementation of physical barriers such as intrauterine devices (IUDs) and Foley catheter balloons,^[^ ^13^, ^14^ ^]^ hormone therapy by exogenous estrogen administration and sildenafil,^[^ ^15^, ^16^ ^]^ and cell therapy such as colony‐stimulating factor, growth factors, and stem cells.^[^ ^17^, ^18^ ^]^ However, the clinical use of these strategies remains problematic due to complications such as recurrent postoperative adhesions, notable disparities in hormonal responses, and secondary infections.^[^ ^19^, ^20^, ^21^ ^]^ These effects are due to the fact that systemic administration of drugs or hormones not only fails to achieve adequate local concentrations in the endometrium, but also increases the risk of systemic hormone imbalances.^[^ ^22^ ^]^ In addition, stem cell therapy, despite its powerful effect on tissue regeneration, faces challenges in clinical use for endometrial repair, such as a low engraftment rate, limited sources, tumor formation, storage, and transportation.^[^ ^23^ ^]^ Therefore, it is imperative to develop effective therapeutic interventions to promote endometrial growth and receptivity, ameliorate endometrial fibrosis, and facilitate the restoration of the damaged endometrium that ultimately reverses the poor pregnancy outcomes of patients with IUA.

With advancements in biological tissue engineering and regenerative medicine, the utilization of biomaterials in endometrial restoration has been investigated.^[^ ^24^, ^25^, ^26^, ^27^ ^]^ Bioactive‐molecule‐mediated tissue repair engineering shows reparative functions through implantation of implanting bioactive scaffolds that have been modified or infused with bioactive factors into tissue defects.^[^ ^28^ ^]^ Among these materials, tissue/organ‐derived extracellular matrix (ECM) materials retain internal structures and cell interactions that closely resemble physiological states that can exhibit physicochemical properties that mimic those of natural tissues. These ECM materials provide abundant extracellular matrix components for resident cells, allowing for the transmission of signaling molecules to deliver their therapeutic effects.^[^ ^29^, ^30^ ^]^ Therefore, the ECM acts as a sophisticated and tissue‐specific structural arrangement for cell adhesion and tissue integrity. Moreover, the ECM plays a pivotal role in tissue homeostasis, immune responses, angiogenesis, and tissue repair.^[^ ^31^ ^]^ Decellularized ECM effectively maintains the native micro/nano architecture of the original tissue or organ, creating an advantageous microenvironment that facilitates cell adhesion, growth, and proliferation.^[^ ^32^ ^]^ Currently, ECM‐related approaches for endometrial restoration include the utilization of stem cell‐ and growth factor‐loaded ECM hydrogels, as well as ECM scaffolds extracted from various sources such as the endometrium, uterus, amnion, and bladder.^[^ ^33^, ^34^, ^35^, ^36^, ^37^, ^38^, ^39^ ^]^ Despite considerable progress achieved in the use of decellularized ECM for endometrial repair, several limitations still exist: 1) ECM materials need to be equipped with exogenous stem cells, which cannot mobilize in situ uterine cells for repair, and the clinical use of stem cells may have risks such as tumor formation. 2) It is difficult for existing ECM materials to retain active growth factors due to preparation methods and other reasons, so it is necessary to carry exogenous cytokines for tissue repair. 3) The retention of hydrogel materials in the uterine cavity remains challenging, limiting their further clinical application and widespread adoption. Therefore, it is essential to develop regenerated living functional materials capable of actively mobilize the migration of in situ endometrial cells and providing them with a nutritive microenvironment.

The microenvironment of endometrial repair is a dynamic and complex regulated system that provides a crucial supporting framework for internal cell migration, proliferation, differentiation, and interaction through signaling molecules and growth factors.^[^ ^40^, ^41^ ^]^ In response to injury, endogenous cells migrate to the damaged area under the guidance of microenvironmental chemokines and growth factors such as vascular endothelial growth factor (VEGF), platelet derived growth factor (PDGF), tumor necrosis factor alpha (TNF‐α), fibroblast growth factor (FGF), and insulin‐like growth factor 1 (IGF‐1) thus contributing to the repair and regeneration of tissues such as uterus.^[^ ^42^, ^43^, ^44^, ^45^, ^46^ ^]^ Therefore, the recruitment of endogenous cells, and the provision of in situ regenerative treatments present a promising therapeutic approach for the repair of the endometrium.^[^ ^47^ ^]^ Considering these factors, it is important to design and construct an ECM material that precisely localizes within the endometrium and releases bioactive substances continuously to facilitate endometrial restoration and regeneration.

**Scheme 1:**
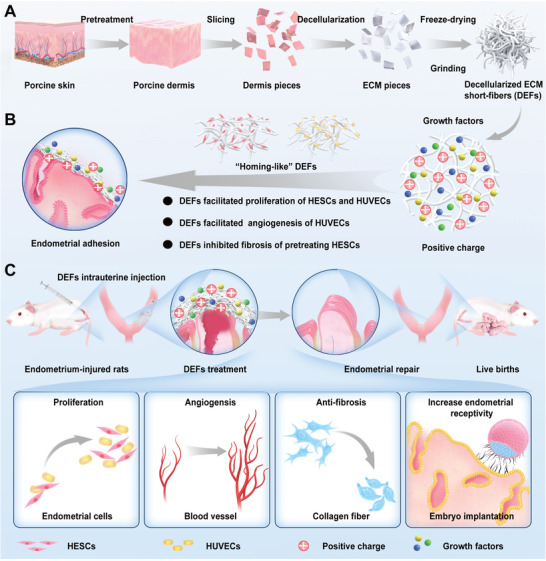
DEFs for endometrium recovery toward efficient live births in rats. Inspired by the cellular homing effect, injectable “homing‐like” bioactive decellularized extracellular matrix short‐fibers (DEFs) of porcine skin origin were innovatively designed for endometrial and fertility restoration. The DEFs rich in bioactive growth factors create a nourishing microenvironment for endometrial recovery while attracting and recruiting endogenous endometrial cells for in situ regeneration. In vivo, DEFs could decrease endometrial collagen deposition and promote endometrial repair endometrial angiogenesis by releasing growth factors. Therefore, the bioactive DEFs could efficiently improve endometrial receptivity, promote endometrial repair, and achieve efficient live births in endometrium‐injured rats.

The concept of “homing” was first introduced by Gallation in 1983,^[^ ^48^ ^]^ which describes the process of circulating lymphocytes returning back to the lymphoid tissues of their origin (home), such as lymph nodes. Inspired by the principles underlying this phenomenon, the present report has developed an innovative method for creating injectable decellularized extracellular matrix short fibers (DEFs) that can effectively attract endometrial cells, enabling cell adhesion, spreading, and proliferation on their surface. DEFs are rich in bioactive growth factors, which creates a nourishing microenvironment for endometrial recovery while attracting and recruiting endogenous endometrial cells for in situ regeneration. Initially, DEFs formed stable bonds with the surface of endometrial cells via electrostatic interactions after injection into the uterine cavity, which acted as a physical barrier to prevent intrauterine adhesions. Subsequently, DEFs effectively facilitated the proliferation and angiogenesis of human primary endometrial stromal cells (HESCs) and human umbilical vein endothelial cells (HUVECs), and inhibited fibrosis of pretreated HESCs. Next, DEFs could decrease endometrial collagen deposition and promote endometrial repair and endometrial angiogenesis by releasing growth factors. Therefore, bioactive DEFs could efficiently improve endometrial receptivity, promote endometrial repair, and achieve efficient live births in endometrium‐injured rats (**Scheme** **1**).

## 2 Results and Discussion

### 2.1 Abnormal Molecular Alterations in Patients with IUA

**Figure 1:**
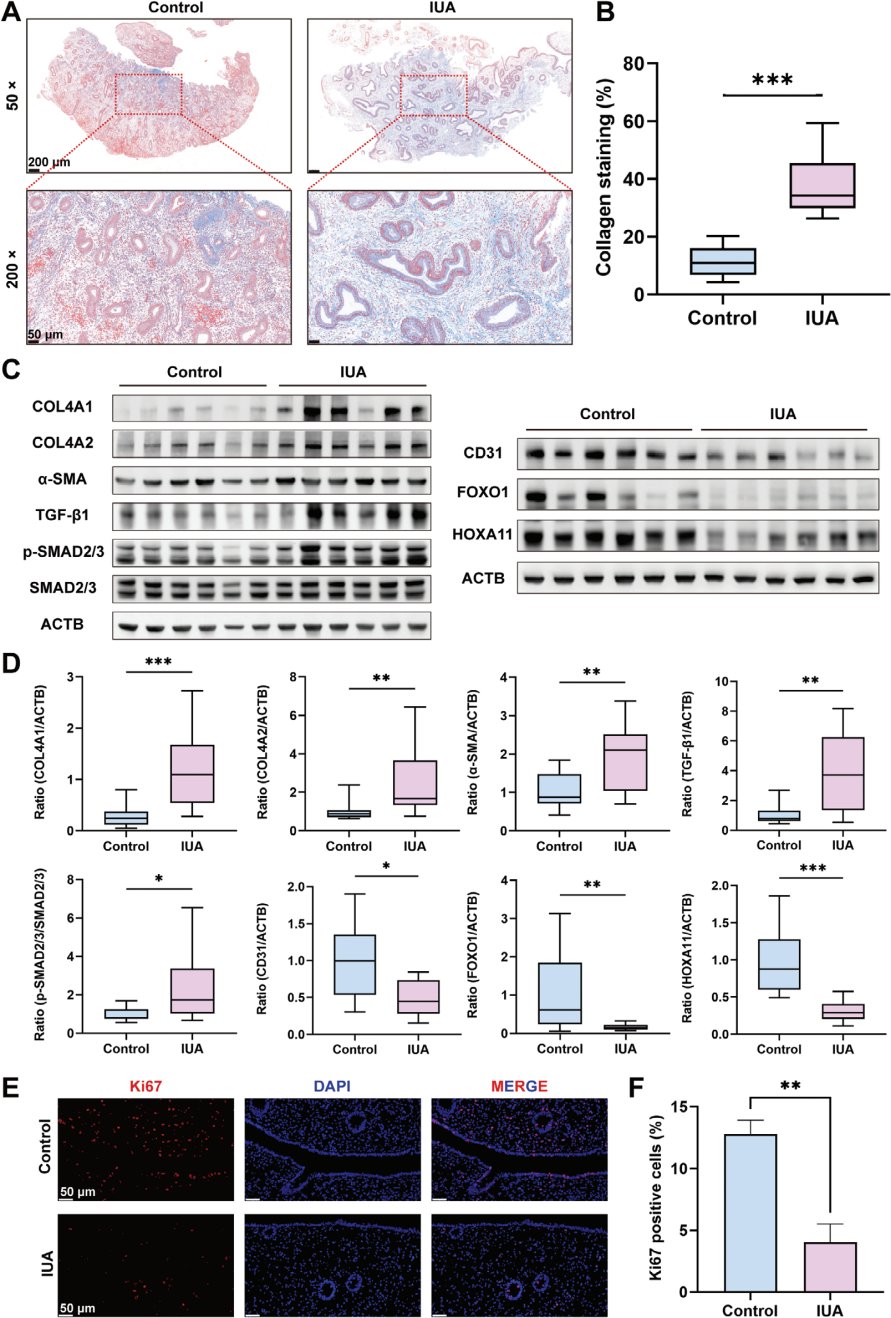
Abnormal molecular alterations in the endometrium of patients with IUA. A) Masson's trichrome staining was used to evaluate endometrial fibrosis (blue, consistent with collagen formation) (× 50 scale bar  =  200 µm and × 200 scale bar  =  50 µm). B) Quantification of collagen protein expression levels (*n* = 20 per group, Student's t test. Data are reported as mean ± SEM, ^***^ *p* < 0.001). C) Western blotting was used to assess the protein expression of markers of fibrosis (COL4A1, COL4A2, α‐SMA, TGF‐β1, p‐SMAD2/3), angiogenesis (CD31), and endometrial receptivity (FOXO1 and HOXA11). D) Relative protein level expression relative to ACTB as a normalization control (*n* = 12 per group, Student's t test. Data are reported as mean ± SEM, ^*^ *p* < 0.05, ^**^ *p* < 0.01, ^***^ *p* < 0.001). E) Immunofluorescence staining of Ki67 (red) (scale bar  =  50 µm). F) Quantification of Ki67 (*n* = 3 per group, Student's t test. Data are reported as mean ± SEM, ^**^ *p* < 0.01).

To explore the molecular alterations occurring in the endometrium of patients with IUA, we conducted a systematic investigation to evaluate the potential changes in fibrosis, angiogenesis, endometrial receptivity, and proliferation compared to those of control subjects. Masson's trichrome staining showed that collagen deposition in the endometrium of the patients with IUA was significantly higher than that of the controls (**Figure** **1**A,B). Consistently, we detected upregulated protein expression of fibrosis‐related markers, such as collagen type IV alpha 1 (COL4A1), COLA4A2, α‐smooth muscle actin (α‐SMA), transforming growth factor beta 1 (TGF‐β1), and phosphorylated SMAD2/3 (p‐SMAD2/3) in the endometrium of the patients with IUAs. Moreover, the protein expression of the angiogenic molecule CD31, as well as the endometrial receptivity markers forkhead box protein O1 (FOXO1), and homeobox protein Hox‐A11 (HOXA11), showed significant decreases (Figure 1C,D). Furthermore, we identified significantly impaired endometrial proliferation in the patients with IUA through Ki67 immunofluorescence (Figure 1E,F). In summary, the endometrium of the patients with IUA showed elevated fibrosis, diminished angiogenesis, decreased endometrial, and impaired proliferation and receptivity.

### 2.2 Fabrication and Characterization of DEFs

**Figure 2:**
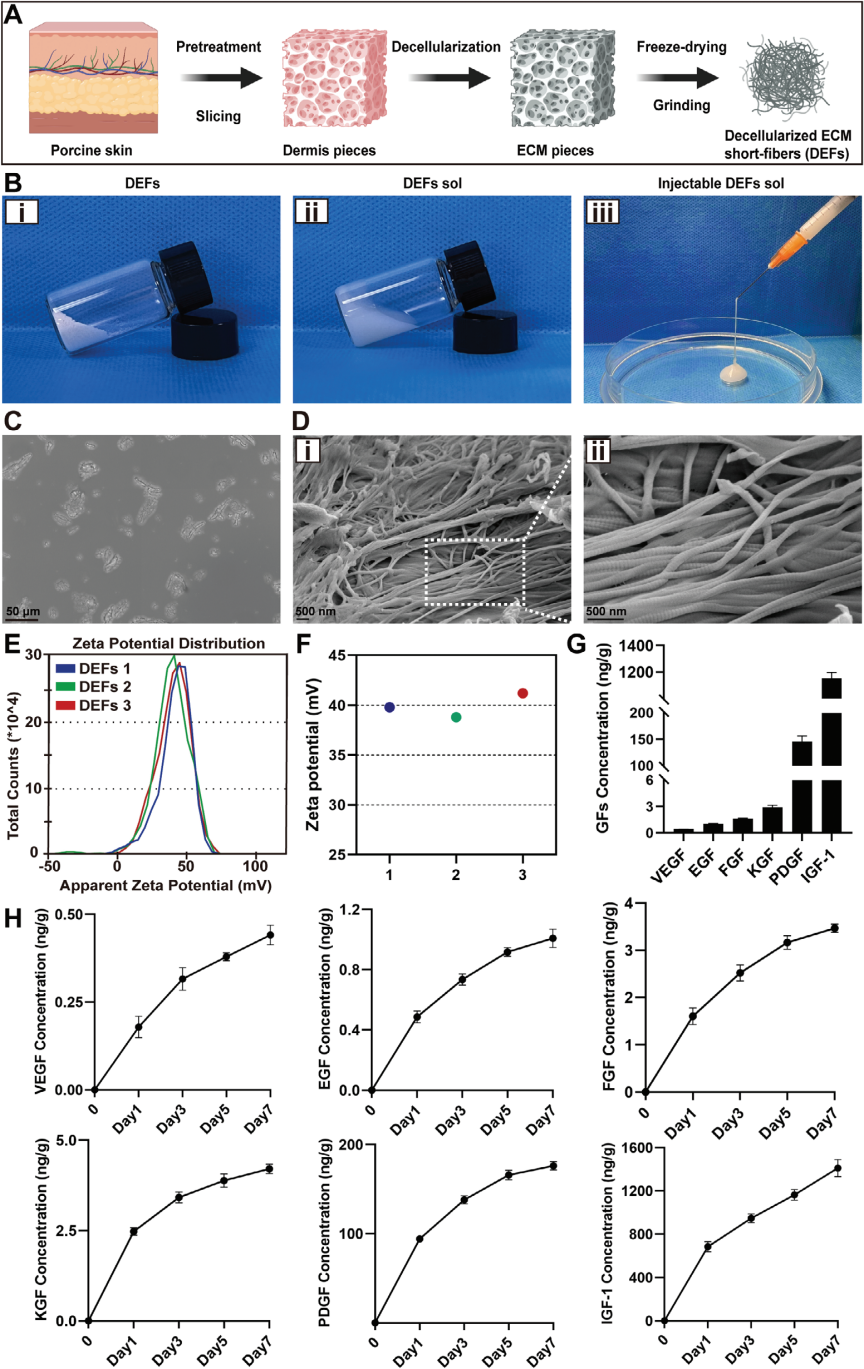
Fabrication and characterization of DEFs. A) The DEFs production process. B) Photograph of the DEFs with different states. i) DEFs in powdered form. ii,iii) Injectable DEFs sol. C) Optical microscope image of DEFs (scale bar  =  50 µm). D) SEM images of DEFs (scale bar  =  500 nm). E,F) Zeta potential of DEFs (*n* = 3). G) Growth factors concentration in the DEFs leaching solution (*n* = 9 per group. Data are reported as mean ± SEM). H) Release curves of VEGF, EGF, FGF, KGF, PDGF, IGF‐1 in DEFs (*n* = 3 per group. Data are reported as mean ± SEM).

**Figure 3:**
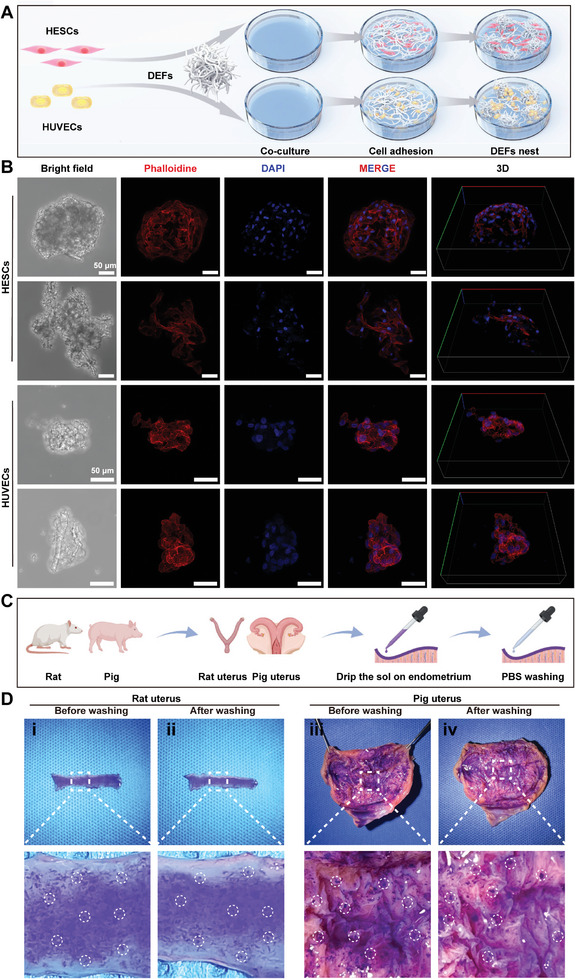
DEFs adhesion detection. A,B) DEFs were cocultured with HESCs and HUVECs, respectively, and the cells were stained with phalloidin (red) (scale bar  =  50 µm). C,D) Adhesion of crystal violet stained DEFs to isolated uteri of rats and pigs, respectively. Some DEFs were circled with white dashed lines as examples. The uteri were perpendicular to the horizontal plane. i,ii) Stained DEFs adhered to the surface of isolated rat endometrium ((i) before PBS washing, (ii) after PBS washing) iii,iv) Stained DEFs adhered to the surface of isolated pig endometrium ((iii) before PBS washing, (iv) after PBS washing).

We used a detailed double decellularization technique to obtain DEFs from porcine skin, as outlined in the methodology (**Figure** **2**A). Next, white powder‐like DEFs were mixed with PBS and guar gum to create an injectable DEFs sol (Figure 2B). As shown in Figure 2C,D, DEFs are micron‐scale short fibers composed of randomly oriented nanoscale filaments. Each filament has a uniform and smooth surface with a micro‐nano structure conducive to cell attachment. The special fibrous structure mimics the natural scaffold of the endometrial ECM, serving as a supportive environment for cells and promoting tissue regeneration. Furthermore, the DEFs display a positive surface charge, with a zeta potential value of ≈39.93 ± 0.70 mV, facilitating the attachment of electronegative endometrial cells (Figure 2E,F). Enzyme‐linked immunosorbent assays (ELISAs) were performed to measure the levels of growth factors in the DEFs: VEGF (0.43 ± 0.02 ng g^−1^), epidermal growth factor (EGF, 1.03 ± 0.04 ng g^−1^), FGF (1.65 ± 0.04 ng g^−1^), keratinocyte growth factor (KGF, 2.90 ± 0.27 ng g^−1^), PDGF (145.74 ± 10.44 ng g^−1^), and IGF‐1 (1150.98 ± 45.14 ng g^−1^) (Figure 2G). These growth factors have been reported to play vital roles in cell proliferation, angiogenesis, and inhibition of cell fibrosis.^[^ ^49^, ^50^, ^51^, ^52^, ^53^, ^54^ ^]^ In order to understand the release behaviors of growth factors from DEFs under physiological condition, we further examined growth factors concentrations after DEFs were immersed in PBS at 37 °C for different time. As demonstrated in **Figure** **3**H, the release curve indicated that the DEFs could provide sustainable release of the growth factors. Furthermore, they act as chemokines to attract and recruit endogenous cells to perform in situ repair in the way of cell homing.

### 2.3 DEFs “Homing‐Like” Effect

To evaluate the adhesive capabilities of DEFs, we cocultured DEFs with HESCs and HUVECs respectively. Through immunofluorescence staining of phalloidin, we observed that both types of cells displayed strong adherence, growth, and stretching capabilities on the DEFs. DEFs exhibited a distinctive clustering pattern, resembling a nest shape. This phenomenon, resembling a “homing‐like” effect, facilitates the effective localization of DEFs in the endometrium (Figure 3A,B). Then, we used isolated uterine samples from rats and pigs to further evaluate the endometrial adhesion of DEFs (Figure 3C). We applied crystal violet stained DEFs sol onto the endometrial surface and observed strong adhesion of DEFs to the endometrium. Neither holding the uterus perpendicular to the horizontal plane nor flushing with PBS altered the amount of DEFs on the endometrial surface (Figure 3D). These results suggest that our bioactive injectable “homing‐like” DEFs can adhere to the endometrium and present a kind of “homing‐like” effect.

### 2.4 In Vitro Assessment of DEFs

To evaluate the effect of DEFs on the biocompatibility and biological function of endometrial cells, we used HESCs and HUVECs for in vitro cytology experiments.

#### 2.4.1 DEFs Promote HESCs Proliferation and Inhibit Fibrosis

**Figure 4:**
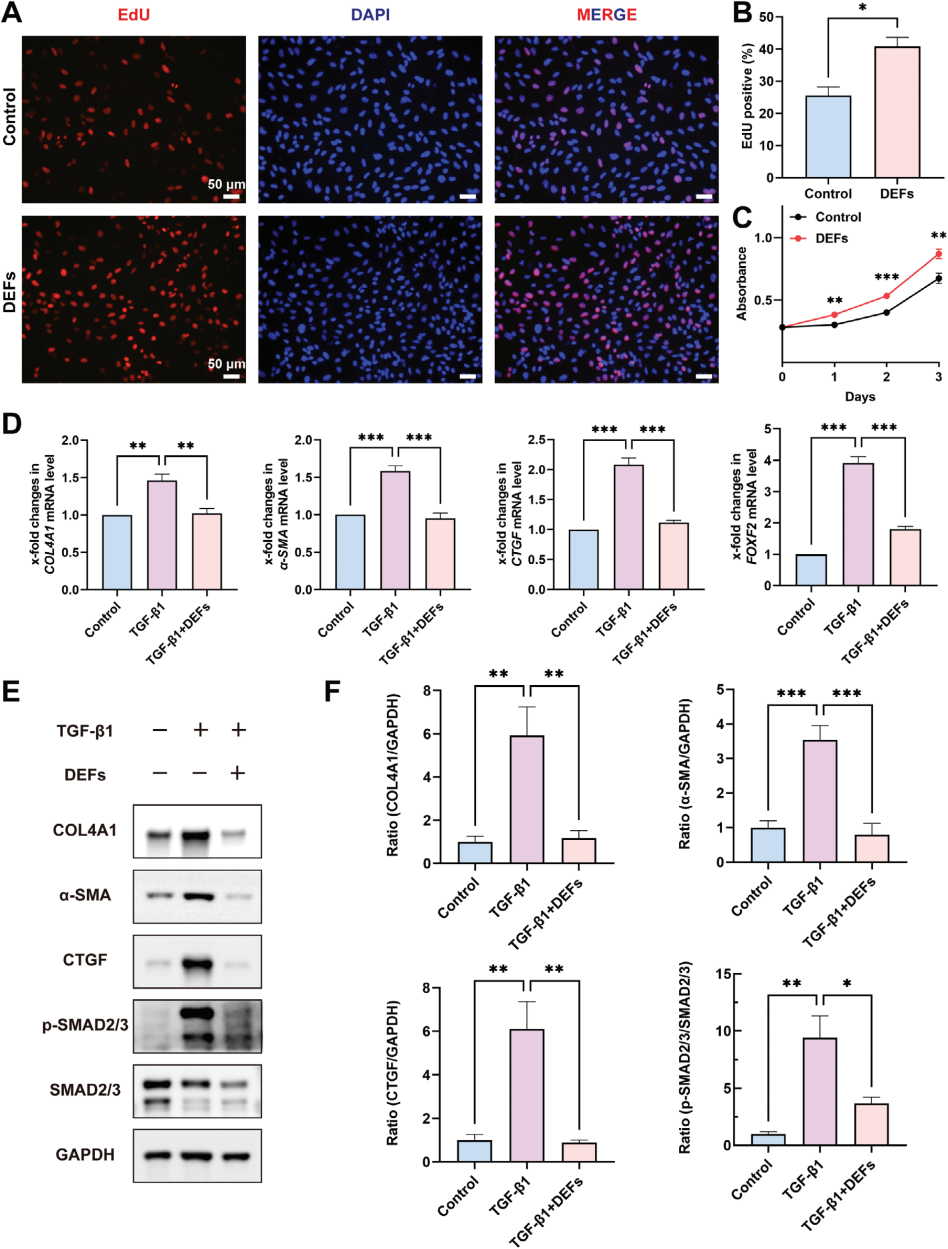
DEFs promote HESCs proliferation and inhibited fibrosis. A) Immunofluorescence staining of EdU (red) in HESCs (scale bar  =  50 µm). B) The EdU positive rate in the two groups (*n* = 6 per group, Student's t test. Data are reported as mean ± SEM, ^*^ *p* < 0.05). C) CCK‐8 tests of HESCs on Day 1, Day 2 and Day 3 (*n* = 6 per group, Student's t test. Data are reported as mean ± SEM, ^**^ *p* < 0.01, ^***^ *p* < 0.001). D) A qRT‐PCR approach was used to assess the mRNA expression of *COL4A1*, *α‐SMA*, *FOXF2* and *CTGF*, with *L19* serving as a normalization control (*n* = 6 per group, one‐way ANOVA, Bonferroni test. Data are reported as mean ± SEM, ^**^ *p* < 0.01, ^***^ *p* < 0.001). E) Western blotting was used to assess the protein expression of markers of fibrosis (COL4A1, α‐SMA, CTGF and p‐SMAD2/3). F) Relative protein level expression relative to *GAPDH* as a normalization control (*n* = 6 per group, one‐way ANOVA, Bonferroni test. Data are reported as mean ± SEM, ^*^ *p* < 0.05, ^**^ *p* < 0.01, ^***^ *p* < 0.001).

The purity of HESCs was distinguished from that of human endometrial epithelial cells by differential expression of the stroma‐specific marker vimentin (VIM) and the epithelia‐specific marker cytokeratin 18 (CK18). Immunofluorescence showed that the purity of HESCs was >95% and qualified for subsequent experiments (Figure S1, Supporting Information). After DEFs were cocultured with HESCs for 24 h, the 5‐ethylnyl‐2′‐deoxyuridine (EdU)‐positive rate in the DEFs group was nearly twice that of the control group (**Figure** **4**A,B). Moreover, we detected cell counting kit‐8 (CCK‐8) levels on HESCs co‐cultured with DEFs on a daily basis. As shown in Figure 4C, the absorbance of the DEFs co‐cultured group was consistently higher than that of the control group throughout the experiment. TGF‐β1 induces fibroblast‐to‐myofibroblast differentiation, thereby contributing to fibrosis.^[^ ^55^ ^]^ To validate the potential anti‐fibrotic effects of DEFs, we established an in vitro fibrosis model using TGF‐β1. After stimulation of the HESCs with TGF‐β1 (10 ng mL^−1^) for 48 h, quantitative real‐time PCR (qRT‐PCR) showed that the mRNA expression of the fibrosis marker molecules *COL4A1*, *α‐SMA*, connective tissue growth factor (*CTGF*), and forkhead box F2 (*FOXF2*) increased significantly. However, when the HESCs were treated with DEFs for 24 h after the 48 h of TGF‐β1 stimulation, a significant reduction in the mRNA levels of these fibrosis markers was observed (Figure 4D). Western blotting results corroborate these findings, and the phosphorylation levels of SMAD2/3 were correspondingly altered (Figure 4E,F).

#### 2.4.2 DEFs Promote HUVECs Proliferation, Migration, and Tube Formation

**Figure 5:**
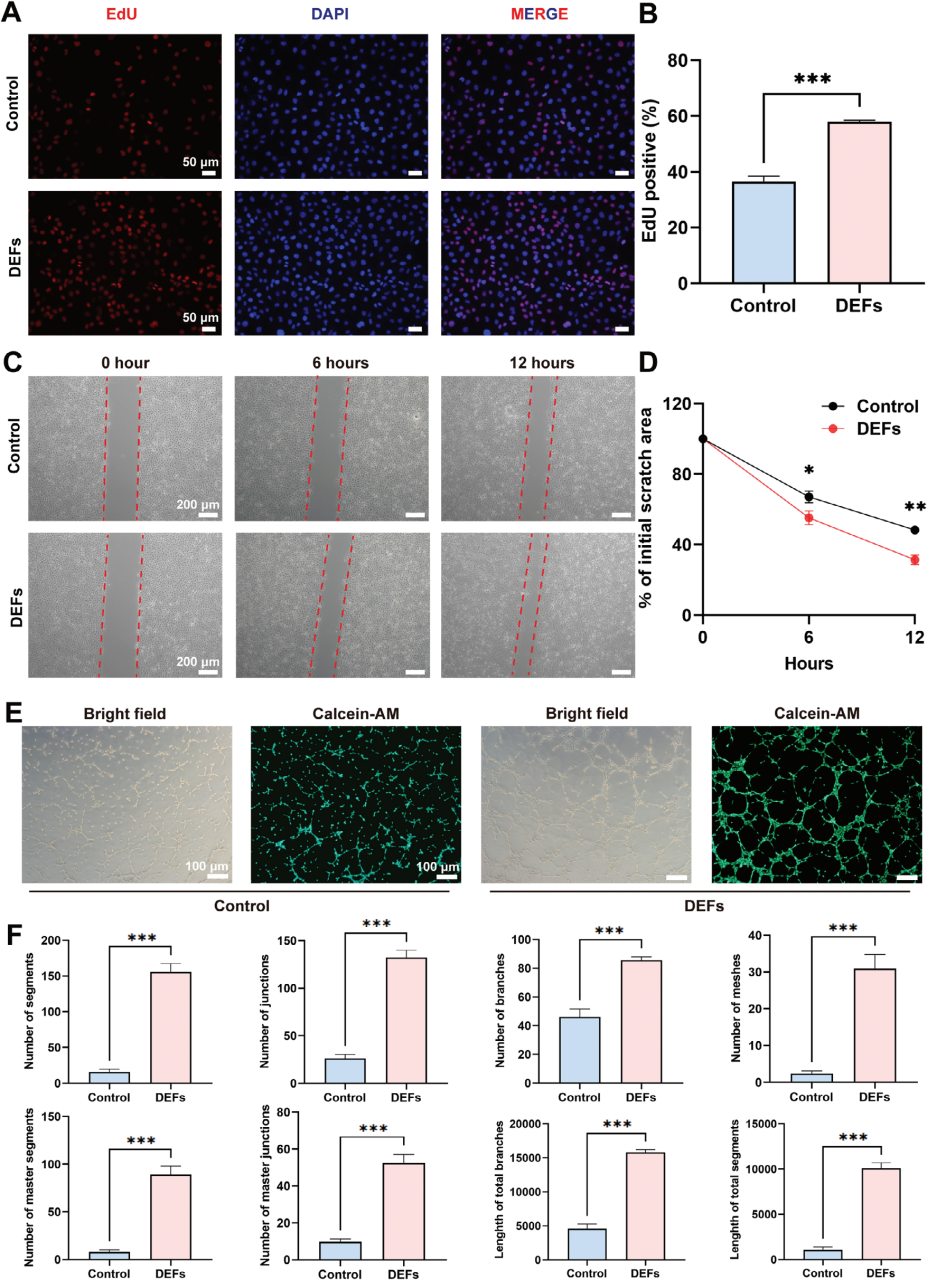
DEFs promote HUVECs proliferation, migration, and tube formation. A) Immunofluorescence staining of EdU (red) in HUVECs (scale bar  =  50 µm). B) EdU‐positive rate in the two groups (*n* = 6 per group, Student's t test. Data are reported as mean ± SEM, ^***^ *p* < 0.001). C) Representative bright‐field images of HUVECs after treatment with DEFs for 0, 6, and 12 h (scale bar  =  200 µm). D) Quantitative results of wound closure rates at different time points (*n* = 6 per group, Student's t test. Data are reported as mean ± SEM, ^*^ *p* < 0.05, ^**^ *p* < 0.01). E) Images of the in vitro formed tubes in HUVECs (scale bar  =  100 µm). F) Quantitative data of tube intensity in different evaluation indices (*n* = 6 per group, Student's t test. Data are reported as mean ± SEM, ^***^ *p* < 0.001).

Angiogenesis is the process by which endothelial cells proliferate, migrate, and form tubules to create new blood vessels that support tissue repair. Here, we evaluated the effect of DEFs on the proliferative ability of HUVECs by EdU assays. The EdU‐positive rate of the DEFs‐treated group was nearly 58.01 ± 0.51%, which was 1.5 times that of the control group (36.58 ± 1.89%) (**Figure** **5**A,B). We also evaluated the effect of DEFs on the migration and angiogenesis of HUVECs in vitro. Wound healing experiments showed that the ECM group healed 70% at 12 h compared to 50% in the control group indicating better cell migration in the DEFs‐treated group (Figure 5C,D). For angiogenic evaluation, we cultured HUVECs on Matrigel with serum‐free DMEM/F12 and DMEM/F12 containing DEFs for 6 h and observed tube formation by Calcein‐AM staining. An angiogenesis assay indicated that DEFs can promote tube formation (Figure 5E,F).

In summary, DEFs have good biocompatibility, can promote the proliferation and angiogenesis of HESCs and HUVECs, and inhibit fibrosis in pretreated HESCs.

### 2.5 In Vivo Assessment of DEFs Therapeutic Effects

We established an in vivo model of endometrial damage in SD‐rats by injecting 95% ethanol into the uterine horn.^[^ ^56^ ^]^ Following the injury, we administered DEFs sol into the uterine cavity and assessed its impact on endometrial regeneration, fibrosis, angiogenesis, endometrial receptivity, and fertility restoration.

#### 2.5.1 DEFs Promote Rat Endometrial Regeneration

**Figure 6:**
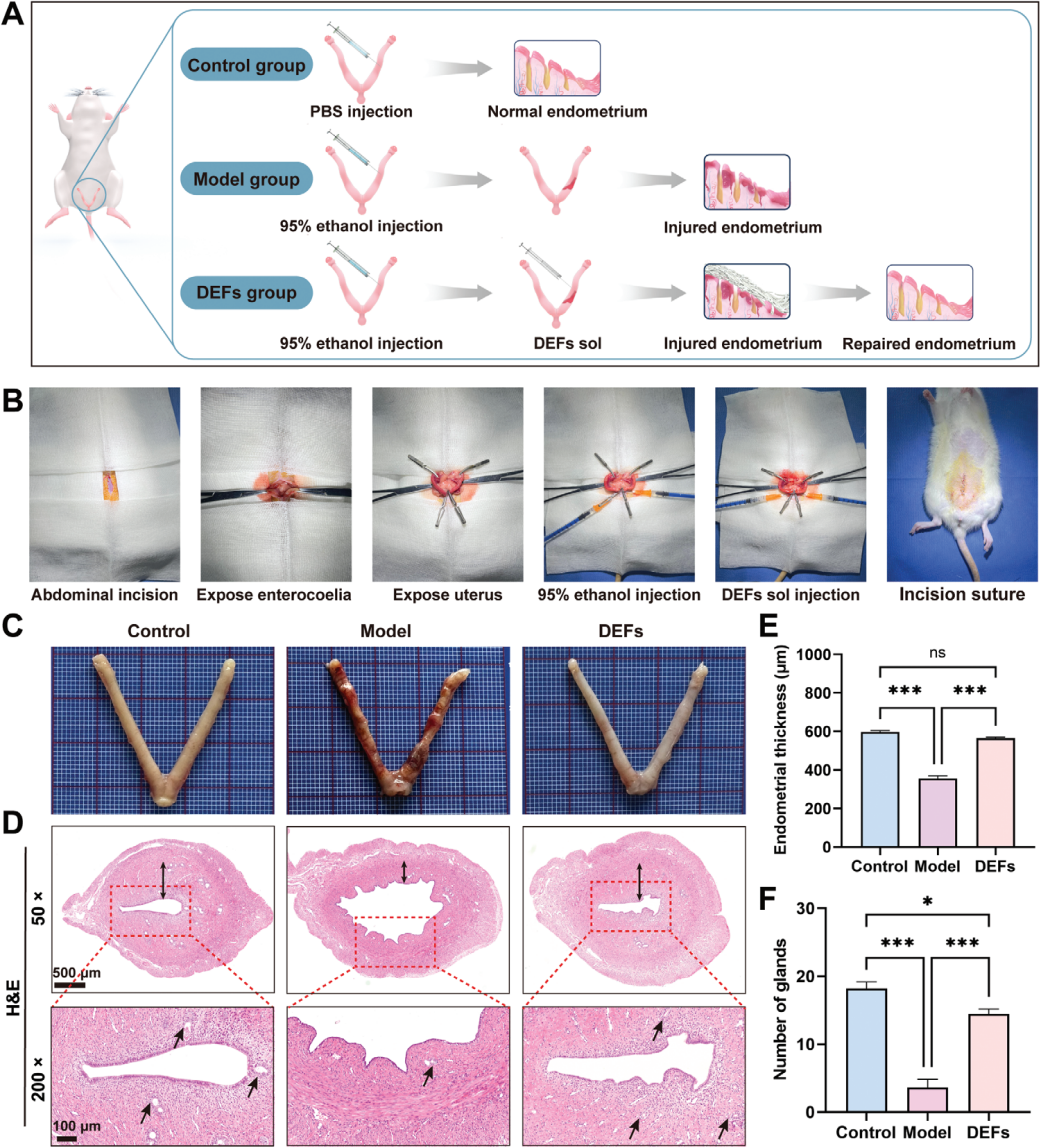
Assessment of treatment‐related changes in uterine morphology. A) Modeling and treatment diagram of rat model. B) Surgical procedures used to generate a rat model of uterine horn damage. Briefly, a mid‐abdominal incision was made to expose the uterine horns, after which 95% ethanol was injected into the uterus. After PBS cleaning, DEFs sol was injected into the uterus. C) Images of the isolated uterus of three groups of rats on the 14th day after surgery. D) Uterine tissue samples from each group were subjected to HE staining (× 50 scale bar  =  500 µm and × 200 scale bar  =  100 µm). With bi‐directional black arrows indicating the endometrial thickness, and unidirectional black arrows indicating the glands. E) Endometrial thicknesses in each group (*n* = 16 per group, one‐way ANOVA, Bonferroni test. Data are reported as mean ± SEM, ^***^ *p* < 0.001, ns means no significance). F) Numbers of glands in each group (*n* = 16 per group, one‐way ANOVA, Bonferroni test. Data are reported as mean ± SEM, ^*^ *p* < 0.05, ^***^ *p* < 0.001).

The female rats were randomly divided into three groups: the control group: sham‐operated rats injected in the uterine horn with PBS; the model group: rats with no treatment after endometrial injury; and the DEFs group: rats with DEFs treatment after endometrial injury (**Figure** **6**A,B). On postoperative day 14, endometrial damage and repair were assessed by appropriate histological analysis. Compared with that of the control group, the damaged uterine tissue in the model group showed a significant thinning of the endometrium with scarce glands and the endometrium was indistinguishable from the myometrium in some areas. In the DEFs group, the stroma and epithelium were well distributed, and the endometrial thickness and gland number increased (Figure 6C,D). Specifically, in the control group, the average endometrial thickness was 597.50 ± 7.07 µm with >18.21 ± 0.96 glands. However, in the model group, the endometrium thickness was only 355.67 ± 14.91 µm and the glands nearly disappeared. In contrast, the average endometrial thickness of the DEFs group was ≈566.46 ± 4.58 µm with an average of 14.46 ± 0.72 glands, which showed the remarkable tissue regenerative capability of the DEFs (Figure 6E,F).

**Figure 7:**
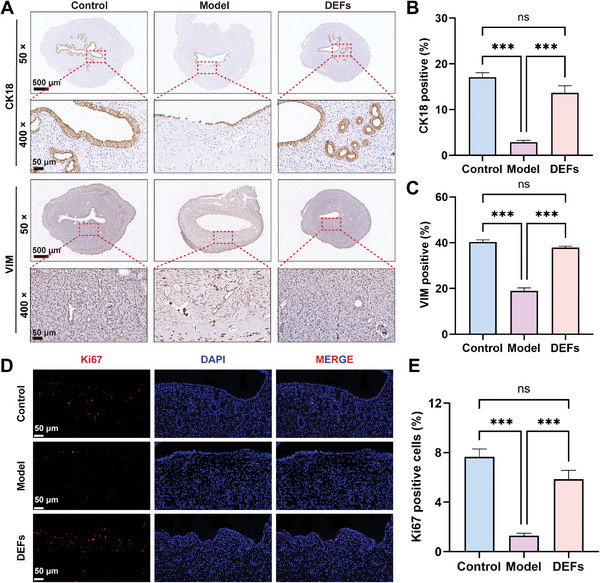
DEFs promote rat endometrial proliferation. A) Immunofluorescence staining of CK18 was used to visualize rat endometrial epithelial cells, while VIM protein staining was used to detect rat endometrial stromal cells (× 50 scale bar  =  500 µm and × 400 scale bar  =  50 µm). B,C) Quantification of CK18 and VIM protein expression levels (*n* = 16 per group, one‐way ANOVA, Bonferroni test. Data are reported as mean ± SEM, ^***^ *p* < 0.001, ns means no significance). D) Immunofluorescence staining of Ki67 (red) (scale bar  =  50 µm). E) Quantification of Ki67 (*n* = 16 per group, one‐way ANOVA, Bonferroni test. Data are reported as mean ± SEM, ^***^ *p* < 0.001, ns means no significance).

Immunohistochemical staining of CK18 and VIM positive cells also suggested that the total number of endometrial epithelial and stromal cells in the model group was significantly reduced, while in the DEFs group the cell numbers almost recovered to the level of the control group (**Figure** **7**A,C). Immunofluorescence staining of Ki67 in the rat endometrium indicated that endometrial proliferation was severely impaired in the model group, while the Ki67 level in the DEFs group was significantly higher than that in the model group (Figure 7D,E). In summary, DEFs could play a role in endometrial repair through the proliferation of endometrial epithelial cells and stromal cells. Local release of chemokines, including VEGF, FGF, and IGF‐1, by DEFs may attract endogenous endometrial cells to homing and promote the proliferation of both endometrial epithelial cells and endometrial stromal cells.

#### 2.5.2 DEFs Reduce Endometrial Collagen Deposition

**Figure 8:**
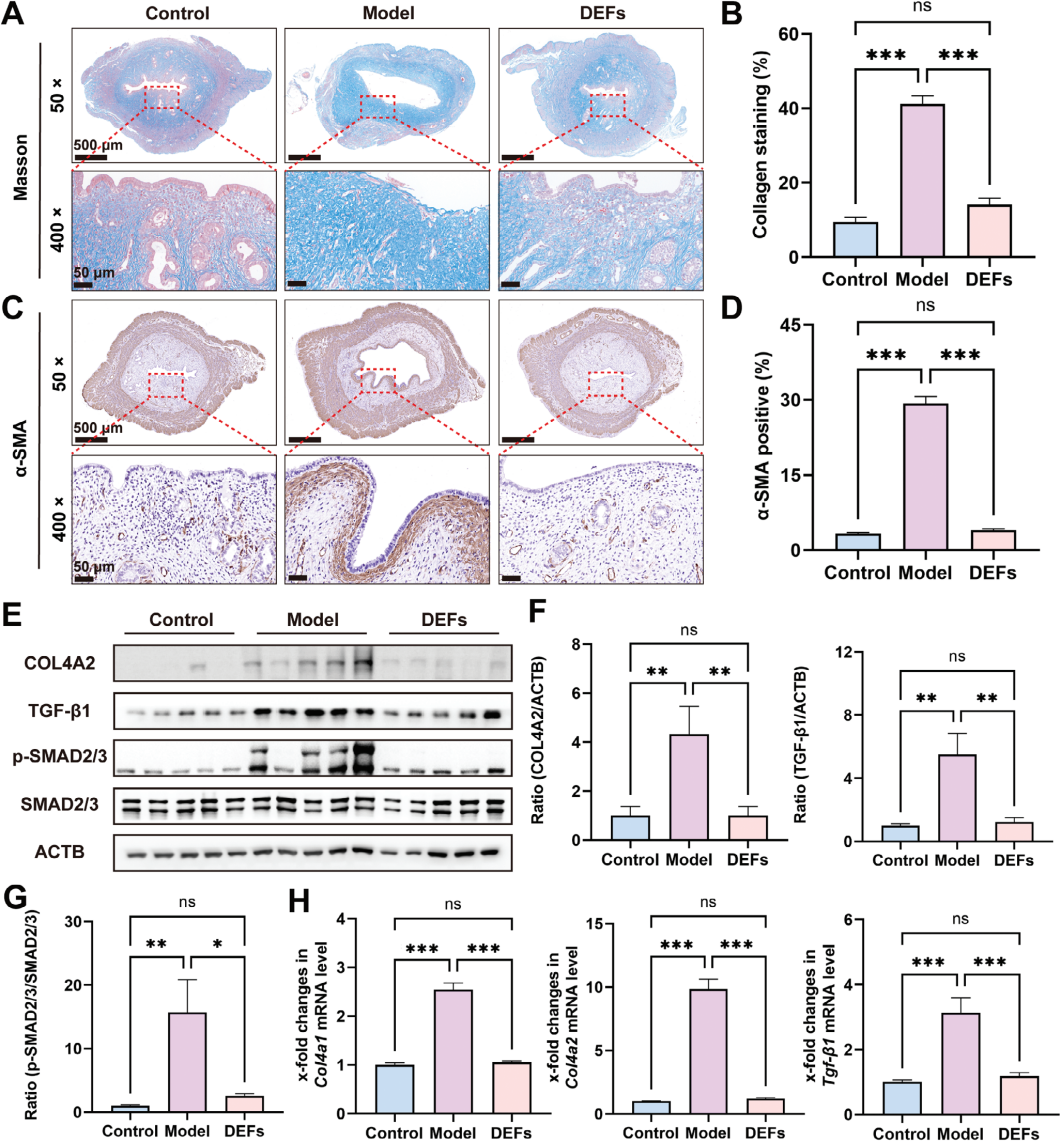
DEFs reduce endometrial collagen deposition. A) Masson's trichrome staining was used to evaluate endometrial fibrosis (blue, consistent with scar formation) (× 50 scale bar  =  500 µm and × 400 scale bar  =  50 µm). B) Quantification of collagen protein expression levels (*n* = 16 per group, one‐way ANOVA, Bonferroni test. Data are reported as mean ± SEM, ^***^ *p* < 0.001, ns means no significance). C) Immunohistochemical staining of α‐SMA to assess endometrial fibrosis (× 50 scale bar  =  500 µm and × 400 scale bar  =  50 µm). D) Quantification of α‐SMA (*n* = 16 per group, one‐way ANOVA, Bonferroni test. Data are reported as mean ± SEM, ^***^ *p* < 0.001, ns means no significance). E) Western blotting was used to assess the protein expression of markers of fibrosis (COL4A2, TGF‐β1, and p‐SMAD2/3). F,G) Relative protein level expression relative to ACTB as a normalization control (*n* = 10 per group, one‐way ANOVA, Bonferroni test. Data are reported as mean ± SEM, ^*^ *p* < 0.05, ^**^ *p* < 0.01, ns means no significance). H) A qRT‐PCR approach was used to assess the mRNA expression of *Col4a1*, *Col4a2*, and *Tgf‐β1*, with *L19* serving as a normalization control (*n* = 10 per group, one‐way ANOVA, Bonferroni test. Data are reported as mean ± SEM, ^***^ *p* < 0.001, ns means no significance).

Through in vitro cell experiments, we observed the inhibitory effect of DEFs on fibrosis in pretreated HESCs. To further evaluate endometrial fibrosis in rats, we conducted Masson's trichrome staining to examine endometrial collagen deposition in the three groups. A significant increase in endometrial collagen deposition was observed in the model group (collagen staining 41.19 ± 2.16%), which was four times higher than that in the control group (collagen staining 9.41 ± 1.31%). In contrast, the DEFs group (collagen staining 14.15 ± 1.70%) exhibited a 13% reduction in collagen deposition compared with the model group (**Figure** **8**A,B). Moreover, we performed α‐SMA immunohistochemical staining on the endometrial tissues of the three groups. The positive rate of the model group was ≈29.28 ± 1.43%, nearly ten times higher than that of the control group (3.37 ± 0.16%) and seven times higher than that of the DEFs group (4.01 ± 0.27%) (Figure 8C,D). Furthermore, we assessed the expression of uterine fibrosis‐associated molecules by qRT‐PCR and Western blotting. The model group displayed significantly elevated mRNA expression of *Col4a1*, *Col4a2*, and *Tgf‐β1*, along with increased protein levels of COL4A2, TGF‐β1, and phosphorylated SMAD2/3, compared to the control group (Figure 8E–G). Notably, the DEFs group showed considerable improvements in both the mRNA and protein levels of these fibrosis‐associated molecules (Figure 8H). These findings collectively demonstrate the effectiveness of DEFs in alleviating endometrial fibrosis and reducing collagen deposition.

#### 2.5.3 DEFs Promote Endometrial Receptivity and Uterine Angiogenesis

**Figure 9:**
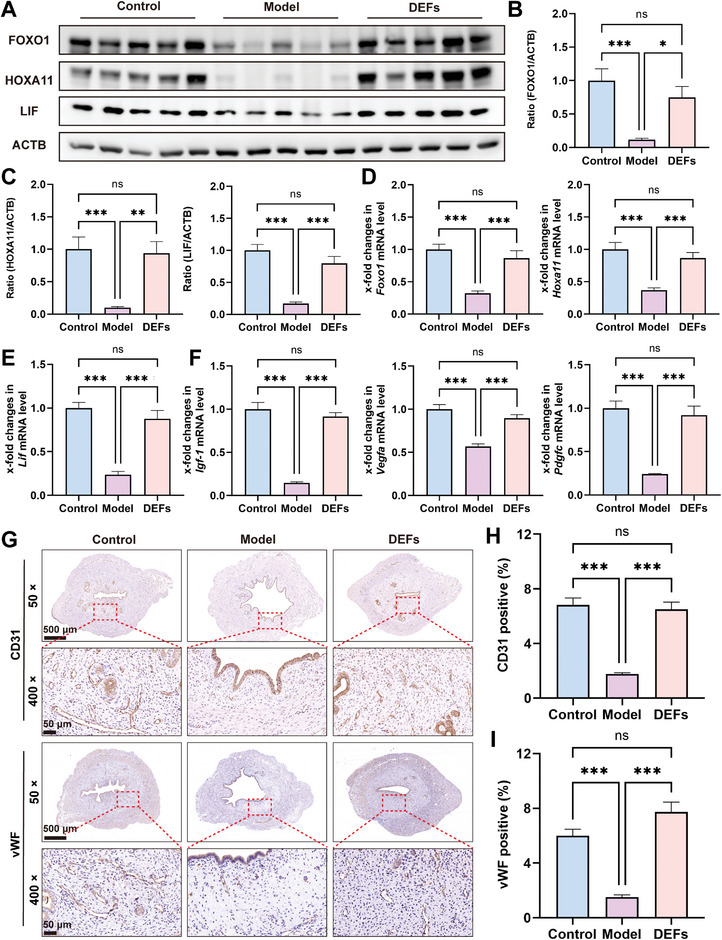
DEFs promote endometrial receptivity and angiogenesis. A) Western blotting was used to assess the protein expression of markers of endometrial receptivity (FOXO1, HOXA11, and LIF). B,C) Relative protein level expression relative to ACTB as a normalization control (*n* = 10 per group, one‐way ANOVA, Bonferroni test. Data are reported as mean ± SEM, ^*^ *p* < 0.05, ^**^ *p* < 0.01, ^***^ *p* < 0.001, ns means no significance). D–F) A qRT‐PCR approach was used to assess the mRNA expression of markers of endometrial receptivity (*Foxo1*, *Hoxa11*, and *Lif*), and angiogenesis (*Igf‐1*, *Vegfa* and *Pdgfc*) with *L19* serving as a normalization control (*n* = 10 per group, one‐way ANOVA, Bonferroni test. Data are reported as mean ± SEM, ^***^ *p* < 0.001, ns means no significance). G) Immunohistochemical staining of CD31‐positive and vWF‐positive endothelial cells in the in the three groups was used to visualize blood vessels (× 50 scale bar  =  500 µm and × 400 scale bar  =  50 µm). H,I) Quantification of CD31 and vWF (*n* = 16 per group, one‐way ANOVA, Bonferroni test. Data are reported as mean ± SEM, ^***^ *p* < 0.001, ns means no significance).

The rat endometrial receptivity was evaluated by the expression of established receptivity marker genes such as FOXO1, HOXA11, and LIF. These factors have been extensively studied and have demonstrated pleiotropic effects on a range of related developmental processes.^[^ ^57^, ^58^, ^59^ ^]^ As shown in **Figure** **9**A–C, the protein levels of FOXO1, HOXA11, and leukemia inhibitory factor (LIF) in the model group were significantly lower than those in the control group, but showed improvement in the DEFs group. The mRNA levels of *Foxo1*, *Hoxa11*, and *Lif* displayed a similar pattern as the protein levels (Figure 9D,E). Additionally, we evaluated the expression of genes involved in angiogenesis and endothelial cell proliferation which can optimize blastocyst implantation by regulating vascular permeability.^[^ ^60^ ^]^ qRT‐PCR results demonstrated decreased mRNA levels of *Igf‐1*, *Vegfa*, and *Pdgfc* in the model group compared with the control group, but a significant recovery was observed in the DEFs group, which were close to the control group level (Figure 9F). Immunohistochemistry staining of the rat uterus showed higher expression of CD31 and vWF in the DEFs group than in the model group, and there was no significant difference between the DEFs group and the control group (Figure 9G–I). The above results indicated that DEFs increased angiogenesis by effectively repairing the endometrium, improving endometrial receptivity, and providing a solid foundation for subsequent embryo implantation and development.

#### 2.5.4 DEFs Enhance Live Births in Rats with Injured Endometrium

**Figure 10:**
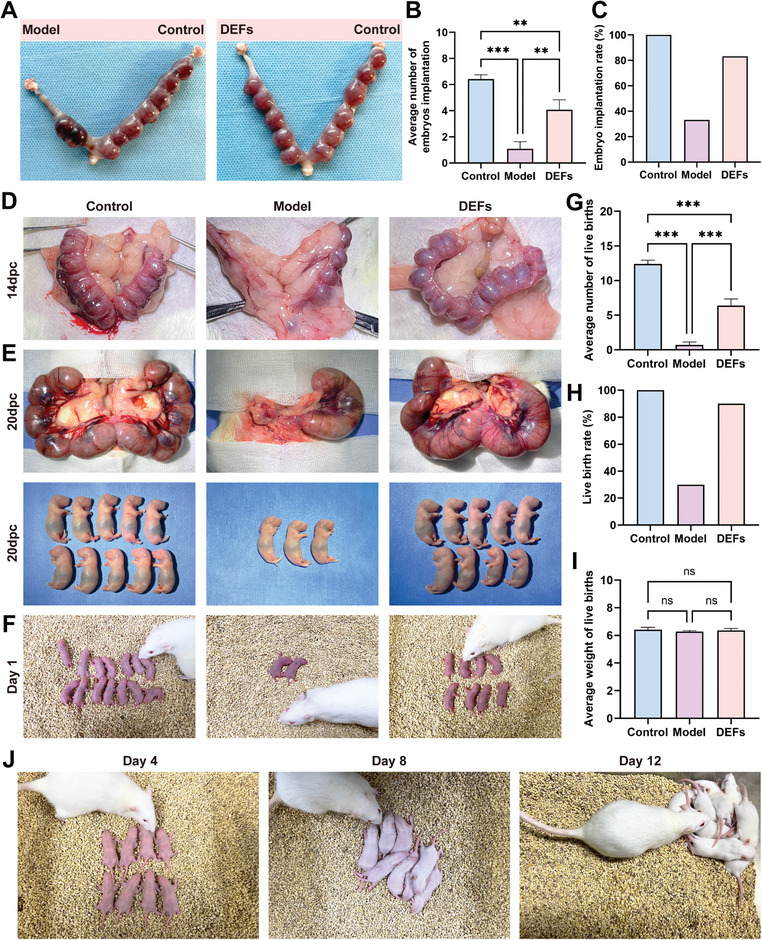
DEFs enhance live births in rats with injured endometrium. A) Embryo implantation in the different groups (the left uterine horn was the model group/DEFs treatment group, and the right uterine horn was the self‐control group) on Day 14 of embryo development. B) Average number of embryos implanted in the three groups (*n* = 12 per group, one‐way ANOVA, Bonferroni test. Data are reported as mean ± SEM, **P < 0.01, ***P < 0.001). C) Embryo implantation rate in the three groups. D) The embryonic development of pregnant rats in the three groups on the 14th day of embryo development. E) The embryonic development of pregnant rats in the three groups on the 20th day of embryo development. F) The live births of pregnant rats in the three groups. G) The average number of live births in the three groups (*n* = 10 per group, one‐way ANOVA, Bonferroni test. Data are reported as mean ± SEM, ^***^ *p* < 0.001). H) Live birth rates in the three groups. I) The weight of fetuses in the three groups (Data are reported as mean ± SEM, ns means no significance). J) Photographic images of newborn rats after feeding for 12 days.

Fertility is considered the gold standard for assessing the recovery of uterine function.^[^ ^61^ ^]^ To assess the healing function of DEFs in rats with severe endometrial injury, we conducted a comprehensive fertility analysis involving embryo implantation, embryonic development, and live births. Initially, we created a self‐control model on the bicornuate uterus of SD rats, designating one side as the control group and the other as the model group or DEFs treatment group post‐modeling. On the 14th day after the operation, SD male rats were paired 1:1 with female rats, and the success of mating was determined by vaginal smears. The rats were euthanized at 14 days post‐coitum (dpc), and the uteri were collected to analyze the number of implanted embryos and implantation rate. The control uterine horn exhibited an average of 6.42 ± 0.34 implanted embryos, while the model side had only 1.08 ± 0.56 implanted embryos (**Figure** **10**A,B). However, the average number of implanted embryos rose to 4.08 ± 0.75 in the DEFs group. Furthermore, the embryo implantation rate in the DEFs group was 83% compared to 33% in the model group (Figure 10C). Statistical results showed that DEFs could significantly increase the number of implanted embryos and the implantation rate of embryos in rats with injured endometrium.

To further investigate late embryo development and live births, we performed bilateral uterine modeling and divided the rats into a control group, a model group and a DEFs group as mentioned above. After a 14‐day recovery period, SD male rats were paired 1:1 with female rats, and successful mating was confirmed by vaginal smears. We examined the development of the three groups of embryos at 14 dpc and 20 dpc. The DEFs group exhibited a similar number of embryos to the control group, while the model group had only three embryos (Figure 10D,E). Subsequently, we observed live births in the three groups at 21–23 days of gestation. The results revealed that the control group had an average of 12.4 ± 0.54 live births, whereas the DEFs group had an average of 6.40 ± 0.96, both significantly higher than that of the model group (0.70 ± 0.42). Moreover, the DEFs group demonstrated an impressive live birth rate of 90%, which was significantly higher than that of the model group (Figure 10F–H). There was no difference in the birth weight of the live‐born offspring of the three groups (Figure 10I). In addition, we monitored the development of offspring in the DEFs group and found their growth to be normal over a 12‐day period (Figure 10J). These findings indicated that intrauterine administration of biologically active DEFs substantially increased the number of live births in rats with uterine damage without adverse effects on the growth and development of the offspring.

### 2.6 Discussion

In recent years, there has been an increase in uterine‐related surgeries, leading to a rise in endometrium factor infertility caused by mechanical injury or chronic inflammation of the endometrium. This has had a significant impact on women of reproductive age.^[^ ^62^ ^]^ Currently, the standard treatment strategies for repairing injured endometrium include balloon stenting and hormone therapy, but the outcomes of these treatments are unsatisfactory. Balloon stenting fails to adequately repair the endometrium, while hormone therapy can result in systemic hormone‐related disorders.^[^ ^63^ ^]^ Despite the potential of stem cell therapy in tissue regeneration, challenges in sourcing appropriate stem cells and concerns regarding their immunogenicity and tumorigenicity have impeded their direct use in endometrial treatment.^[^ ^64^, ^65^ ^]^ Therefore, there is an urgent need to develop novel and effective solutions to restore damaged endometrium promptly.

Biomedical materials are becoming crucial medical tools for tissue repair, and there is growing interest in the development of clinically focused functional biomedical materials for uterine endometrial repair. ECM scaffold materials are composed of structural and functional molecules that characterize natural tissue ECM, playing a crucial role in tissue morphogenesis, differentiation, and homeostasis.^[^ ^66^ ^]^ Decellularized ECM, obtained through processes such as chemical, physical, and enzymatic digestion, removes antigenic components that may cause immune rejection while maintaining the structural integrity and functionality of the ECM and growth factors.^[^ ^67^ ^]^ Consequently, decellularized ECM biomaterials have potential prospects in the field of regenerative medicine.

In order to address endometrial defects, the development of a novel therapeutic material that possesses biological activity and can adapt to the natural tissue structure is crucial. In this study, we constructed a new biomaterial strategy for endometrial repair and the restoration of fertility by intrauterine injection of “homing‐like” bioactive DEFs. The complex micro‐nano structure of DEFs effectively meets the requirements for cell adhesion and growth. Additionally, their positive charge enables them to firmly attach to the surface of the endometrium and slowly release bioactive factors in situ, thereby exerting a sustained effect in damaged tissues. The DEFs could effectively promote endometrial repair and angiogenesis and reduce collagen deposition by releasing bioactive factors, thereby promoting the recovery of fertility to achieve high‐efficiency live births. In vitro experiments had shown that DEFs possessed positively charged surface, facilitating their binding to the endometrial surface through electrostatic interactions, thereby facilitating the repair of damaged endometrium. Additionally, DEFs exhibited high biocompatibility and promoted the proliferation and angiogenesis of HESCs and HUVECs. Moreover, DEFs show the ability to inhibit fibrosis in pretreated HESCs.

Accordingly, we established an in vivo endometrium‐injured model in SD‐rats and treated them with intrauterine injections of DEFs sol. The results of in vivo experiments demonstrated that DEFs could reduce collagen deposition in the endometrium and promote endometrial repair and angiogenesis by releasing growth factors. The observed effects can be attributed to the abundance of growth factors present in DEFs, such as EGF, FGF, KGF, IGF‐1, PDGF, which have been shown in previous studies to be involved in tissue repair and have potential applications in regenerative medicine.^[^ ^49^, ^50^, ^53^ ^]^ Moreover, VEGF and KGF have been found to possess anti‐fibrotic properties.^[^ ^51^, ^68^ ^]^ These beneficial effects of DEFs are likely due to their ability to stimulate cell proliferation while inhibiting fibrosis. Additionally, DEFs demonstrated a significant promotion of angiogenesis, likely due to the release of growth factors (VEGF, PDGF, IGF‐1) that play crucial roles in facilitating endothelial cell proliferation and migration.^[^ ^51^, ^69^, ^70^ ^]^ Growth factors have a pivotal role in repairing and regenerating the endometrium. They serve as chemokines, attracting and mobilizing endogenous endometrial cells to homing and facilitating in situ regeneration. In addition, although in rat experiments, abdominal incision is required to perform intrauterine injection, in clinical settings however, DEFs can be administered noninvasively to treat patients with endometrial damage. DEFs sol can be injected through the cervical canal into the uterine cavity using a soft‐tip catheter under ultrasound guidance, minimizing the risk of re‐injury to the endometrium. The intrauterine injection of DEFs demonstrates promising potential for future clinical applications.

The process of embryo implantation is intricate and influenced by multiple factors. Successful implantation requires high‐quality embryos, optimal endometrial receptivity, and synchronized development of both the embryos and endometrium.^[^ ^4^ ^]^ Endometrial receptivity is regulated by various elements that induce modifications within the uterus to create a supportive environment for blastocyst implantation.^[^ ^71^ ^]^ In addition to facilitating endometrial repair, the utilization of DEFs has further enhanced endometrial receptivity, establishing a favorable microenvironment for subsequent embryo implantation and successful offspring production.

In this study, we have developed a novel biomaterial strategy for endometrial repair and fertility restoration by injecting bioactive DEFs directly into the uterus. However, the composition of these “homing‐like” bioactive DEFs is complex. The aforementioned hypotheses, based on literature survey and analysis, do not provide conclusive evidence on the functioning of DEFs and the specific proteins involved, apart from the identified growth factors. In our future endeavors, our primary aim is to conduct a proteomic analysis of the DEFs to pinpoint the vital proteins responsible for their ability to induce tissue growth. Subsequently, we intend to utilize DEFs modified with fluorescent probes that will enable us to track the localization and actions of these proteins in vivo. Furthermore, we plan to investigate the effects of these chemokines on endogenous endometrial cells in vivo, with a particular focus on their potential to stimulate cell removal or elimination.

## 3 Conclusion

Here, we summarize that this type of DEFs exhibit the following characteristics: 1) The DEFs possess a positively charged surface, allowing them to bind to endometrial cells through electrostatic dipole interactions. This enables them to play a reparative role in damaged endometrium. 2) By acting as a physical barrier, the DEFs help prevent intrauterine adhesions, while the growth factors they release aid in reducing fibrosis and promoting endometrial regeneration and receptivity. 3) The growth factors released by DEFs recruit endogenous cells to homing to promote repair as chemokines. 4) The preparation method of DEFs is simple, feasible, and suitable for mass production, making them applicable for widespread clinical use in the future. 5) The injectability of DEFs can achieve non‐invasive intrauterine injection, which can reduce the risk of endometrial re‐injury. Overall, the local intrauterine administration of injectable DEFs represents a promising therapeutic approach for endometrial repair and fertility enhancement.

## 4 Experimental Section

### Ethical Approval and Human Sample Collection

The study was approved by the Ethics Committee of Ren Ji Hospital, Shanghai Jiao Tong University School of Medicine (No. KY2021‐211‐B).

Forty patients (20 patients with IUA and 20 controls) aged 20–40 years old were enrolled in this study during their visits at the Infertility Consulting Clinic of Center for Reproductive Medicine, Ren Ji Hospital, Shanghai Jiao Tong University School of Medicine. Patients with IUA were diagnosed by hysteroscopy examination by a skilled endoscopist and classified according to the American Fertility Society classification scoring method (The American Fertility Society, 1988).^[^ ^72^ ^]^ The specific inclusion/exclusion criteria are provided in the Supporting Information. Control subjects were women with an endometrial thickness >7 mm (measured preoperatively by ultrasound) and no endometrial lesions found under hysteroscopy. Endometrial biopsy was performed during the late proliferative phase of the menstrual cycle under hysteroscopy, and endometrial tissues from various parts of the uterus (anterior and posterior uterine walls, uterine fundus, adhesion area in patients with IUA) were obtained. In addition to pathological examination, the remaining endometrium was used for experimental studies. Isolated primary human endometrial stromal cells for in vitro experiments were collected from another six healthy women who underwent hysteroscopy examination. Informed consent was obtained from all patients.

### Animal Ethics

Sprague‒Dawley (SD) rats used in this study were purchased from Beijing Vital River Laboratory Animal Technology Co., Ltd. (Beijing, China). All animal experiments conducted conformed with the Guide for the Care and Use of Laboratory Animals (NIH Publications No. 80‐23) and were approved by the Animal Care Committee of Ren Ji Hospital, Shanghai Jiao Tong University School of Medicine (RJ2022‐0829). The animal units were in a temperature (20–26 °C) and humidity (40–70%) controlled facility with a 12 h light/dark cycle (lights on at 6 a.m.), standard sterile laboratory feed and pure water. Vaginal smears were obtained daily at 8–10 a.m.

### Preparation of DEFs

Fresh pigskin (without detectable disease) was selected for DEFs production. First, the epidermis and fat were removed from the porcine skin, leaving the dermis, and freezing the samples at −20 °C after washing with deionized water.

- Disinfection: The dermis was cut into pieces (≈1 cm × 1 cm × 0.5 cm) and then added to aqueous solution A containing 1% sodium hydrogen phosphate and 5% peracetic acid and shaken gently on a stir plate for 3 h at room temperature (RT).
- Softening and homogenization: After the dermis pieces were washed with 0.9% normal saline three times, they were added to another aqueous solution B containing 2% sodium carbonate sodium and 0.2% Triton X‐100 solution and stirred for 3 h at RT. After another wash with 0.9% normal saline, softening dermis pieces were obtained. Then, the dermis pieces were homogenized 30 times with a knife grinder at 8000 rpm.
- Decellularization: The dermis pieces were incubated in aqueous solution C containing 0.1% HEPES, 0.2% disodium edetate and 1% deoxycholate solution and shaken gently on a stir plate at RT overnight. After shaking, the dermis pieces were washed with aqueous solution C ten times and then washed with deionized water three times. The decellularized ECM was obtained after the end.
- Freeze‐drying and grinding: The decellularized ECM was lyophilized using a freeze‐dryer and ground into short‐fibers (product particle size <2 mm). After that, the DEFs were obtained.
- Sterilization: The DEFs were irradiation sterilized (17.6 kGy) and then sealed packaged for further use.
- DEFs sol: The DEFs sol was a mixture of DEFs, PBS and guar gum at a ratio of DEFs: PBS: guar gum = 0.1 g: 1 mL: 0.01 g, and the final concentration of DEFs was 10% (w/v).

### Characterization of DEFs

The morphology and size of the DEFs were observed by a bright‐field microscope (LSM800, Zeiss, Germany). The surface morphology of the DEFs was examined through SEM (FEI Sirion 200, USA). Additionally, the zeta potential of the DEFs was measured using a ZetaSizer Nano ZS (ZNS3600, Malvern Instruments, UK).

### Detection of Active Growth Factors

A mixture of 0.5 g DEFs was ground on ice for 10 min in 4.5 mL of PBS, and the supernatant was carefully collected after centrifugation (1000 rpm for 20 min, RT). Growth factor levels were analyzed using the corresponding ELISA kit (Mlbio, Shanghai, China). Detailed information on the ELISA kits is listed in Table S1 (Supporting Information).

### Growth Factor Release

1 g DEFs immersed in 10 mL of PBS (pH 7.4) in a 15 mL centrifuge tube and were oscillated in a shaker incubator at 37 °C for 7 days. The release medium (1.36 mL) was collected at scheduled time intervals for ELISA kit (Mlbio, Shanghai, China) analysis and replaced with an isopyknic PBS at scheduled time intervals (Day 1, Day 3, Day 5, Day 7). Detailed information on the ELISA kits is listed in Table S1 (Supporting Information).

### Isolation of Primary Human Endometrial Stromal Cells

For isolation of primary human endometrial stromal cells (HESCs) from endometrial biopsy samples, a selective attachment strategy was employed based on previous methods with some modifications.^[^ ^73^ ^]^ Briefly, the specimens were washed with DMEM/F12 medium with 10% (v/v) fetal bovine serum (FBS) (Gibco, Grand Island, NY) and 1% (v/v) penicillin‒streptomycin‐neomycin (Gibco, Grand Island, NY) to remove excess red blood cells, followed by digestion with 0.1% (w/v) collagenase type I (Sigma–Aldrich, St. Louis, MO) for 40 min at 150 rpm and 0.1% (w/v) deoxyribonuclease I (Sigma–Aldrich, St. Louis, MO) for 15 min without shaking. The digested epithelial/stromal mixtures were filtered through sequential 180 and 40 µm cell strainers. HESCs that passed through the 40 µm strainer were resuspended in DMEM/F12 medium, and their purity was assessed by immunofluorescence of CK18 and VIM. Detailed information on the antibodies is listed in Table S2 (Supporting Information).

### DEFs Adhesion Detection (with Cells)

HESCs and HUVECS were digested respectively, seeded to 10 cm cell culture dish (non‐treated surface), and co‐cultured with 10% (w/v) DEFs using DMEM/F12 medium with 10% (v/v) FBS. After 24 h, cell supernatant was transferred to 15 mL centrifuge tube and centrifuged for 5 min at 1500 rpm. Then the supernatant was discarded leaving the DEFs‐attached cells at the bottom of the tube. After fixation, permeabilization and blocking, cells were incubated with phalloidin (Beyotime, Shanghai, China) for 2 h at room temperature. After staining with DAPI, cells were observed under a fluorescence microscope (LSM800, Zeiss, Germany).

### DEFs Adhesion Detection (with Endometrium)

The stained DEFs sol was a mixture of DEFs, crystal violet solution and guar gum with a ratio of DEFs: 0.1% crystal violet solution: guar gum = 0.1 g: 1 mL: 0.01 g. The stained DEFs sol was dripped till it covered the whole endometrium on ex vivo rat and porcine endometrium surface, respectively. One hour later, the endometrial surface was washed with PBS.

### Cell Viability and Proliferation Assay

10% (w/v) DEFs was prepared using DMEM/F12 medium with 10% (v/v) FBS according to required amount, the suspension was then vortexed vigorously for 5 min and centrifuged for 10 min at 3500 rpm. The supernatant was extracted for further cell culture experiments. Cell proliferation was determined by the uptake of 5‐ethynyl‐2‐deoxyuridine (EdU) into DNA using an EdU Cell Proliferation Kit with Alexa Fluor 594 (Beyotime, Shanghai, China) according to the manufacturer's instructions. Cells were seeded in 96‐well plates and incubated with the DEFs supernatant at 37 °C in a humidified 5% CO_2_ incubator after 24 h. EdU (final concentration 10 µm) was added at the 12th hour of coculture, and the incubation continued for 12 h. Cells were further fixed, permeabilized, blocked and stained according to the instructions. Images were captured by fluorescence microscopy (LSM800, Zeiss, Germany) and analyzed by ImageJ software. Cell viability was determined by a CCK‐8 assay kit (Dojindo, Shanghai, China). The optical density of each well at 450 nm was measured using a microplate reader (Thermo Fisher) after HESCs coculture with DEFs supernatant for 24, 48, and 72 h.

### In Vitro Fibrosis Model

10% (w/v) DEFs was prepared using DMEM/F12 medium with 10% (v/v) FBS according to required amount, the suspension was then vortexed vigorously for 5 min and centrifuged for 10 min at 3500 rpm. The supernatant was extracted for further cell culture experiments. For establishment of an in vitro cell fibrosis model, HESCs were seeded into a six‐well plate and incubated with 10% FBS‐DMEM/F12 medium containing 10 ng mL^−1^ recombinant human TGF‐β1 (PeproTech, USA) at 37 °C for 48 h.^[^ ^51^ ^]^ After treatment with TGF‐β1, the culture medium was replaced with the supernatant of DEFs, and the cells were incubated for another 24 h. RNA and protein were extracted from HESCs for subsequent fibrosis analysis.

### Wound Scratch Assay

For the wound scratch assay, HUVECs were in a culture‐insert (ibidi culture‐insert 2 well, ibidi GmbH, Martinsried, Germany) at a density of 3 × 10^4^ cells per well. After an overnight incubation period for cell attachment, the culture‐insert was carefully removed, and the cells were washed with PBS to eliminate any non‐adherent cells. HUVECs were incubated in the supernatant of DEFs. The scratch area of the HUVECs was photographed after incubation for 0, 6, and 12 h. The scratch healing rate was computed by comparing the closed area and initial wound area.

### Tube formation Assays

10% (w/v) DEFs was prepared using DMEM/F12 medium according to required amount, according to required amount, the suspension was then vortexed vigorously for 5 min and centrifuged for 10 min at 3500 rpm. The supernatant was extracted for further cell culture experiments. 50 µL of Matrigel (ABW, Shanghai, China) was added to each well of 96‐well plates and cultivated in a 37 °C cell incubator for 30 min. Then, 100 µL of the DEFs supernatant containing 2 × 104 HUVECs that had been starved for 24 h was seeded on the surface of the Matrigel. After 6 h, Calcein‐AM (MCE, Shanghai, China) was added to stain HUVECs for 30 min, and images were obtained by fluorescence microscopy. The captured images were analyzed by ImageJ with the Angiogenesis Analyzer plugin^[^ ^74^ ^]^ (http://imagej.nih.gov/ij/macros/toolsets/Angiogenesis%20Analyzer.txt) to quantify different parameters (the specific meanings are provided in the Supporting Information), such as number of segments, number of junctions, number of branches, number of meshes, number of master segments, number of master junctions, length of total branches, and length of total segments. Higher values of these parameters indicate greater quantity and quality of tube formation.

### In Vivo Endometrial Injury Model Construction and Treatment

A rat model of endometrial injury was established by injecting 95% ethanol into the uterus as described previously.^[^ ^56^ ^]^ Female SD rats (200–220 g, 8 weeks old) with normal estrus cycles were randomly divided into three groups: the control group, model group, and DEFs group. Briefly, rats were anesthetized by intraperitoneal injection of 2% sodium pentobarbital (0.3 mL/100 g), and the uterine horns were exposed via an abdominal incision. The control group consisted of sham‐operated rats injected in the uterine horns with 100 µL of PBS. Rats in the model group and DEFs group were injected in the uterine horns with 100 µL of 95% ethanol, and distal portions of the uterus were clamped with vascular clamps to their full capacity and retained for 60 s to induce endometrial injury. The vascular clamps were then released, and the uterine cavity was gently flushed with PBS to remove residual ethanol. For the DEFs group, the DEFs sol was a mixture of DEFs, PBS and guar gum at a ratio of DEFs: PBS: guar gum = 0.1 g: 1 mL: 0.01 g, and the final concentration of DEFs was 10% (w/v). 100 µL of DEFs sol was injected into the uterine cavity after removal of ethanol. Finally, the muscle layer and skin were stitched using 4–0 unabsorbable sutures. Rats were euthanized 14 days after surgery, and the uterine tissues were harvested for downstream analysis.

### Embryo Implantation Test

A self‐control approach was used to evaluate embryo implantation in SD rat models. Specifically, one side of the uterine horn was treated with 95% ethanol or 95% ethanol + DEFs (as previously described for the model group and DEFs group, respectively), while the other side of the uterine horn was used as the control group. On the 14th day after the operation, SD male rats were paired 1:1 with female rats, and the success of mating was determined by vaginal smear. The rats were euthanized at 14 days post‐coitum (dpc), and the uteri were collected to analyze the number of implanted embryos and implantation rate. The assignment of rats for the animal experiment is shown in Table S3 (Supporting Information).

### Embryo Development and Live Birth Test

To further investigate late embryo development and live births, the study performed bilateral uterine modeling and divided the rats into a control group, a model group, and a DEFs group. After a 14‐day recovery period, SD male rats were paired 1:1 with female rats, and successful mating was confirmed by vaginal smear. The development of three groups of embryos was examined at 14 dpc and 20 dpc. The number and rate of live births were observed at approximately 21–23 days of gestation, and the weight of the offspring was measured. The assignment of rats for the animal experiment is shown in Table S4 (Supporting Information).

### Histological and Immunohistochemical Staining

Human endometrium and rat uterine horns were fixed with 4% paraformaldehyde and immersed in wax after gradient dehydration. Tissues were sliced into 10 µm thick sections for subsequent experiments. Tissue slices were subjected to HE and Masson's trichrome staining based on the provided instructions. The distance from the inner layer of the rat endometrium to the myometrium was measured horizontally and vertically in the HE staining images of the three cross‐sections of the uterus, and the average value was calculated. Collagen deposition was analyzed by ImageJ software based on Masson's trichrome staining images. Levels of endometrial fibrosis and angiogenesis were assessed via immunohistochemical staining. The slices were deparaffinized, rehydrated, and incubated with different primary antibodies overnight at 4 °C. On the next day, the slices were incubated with secondary antibodies at room temperature for 1 h, followed by DAB chromogenic reaction and hematoxylin staining. Images were captured by an optical microscope and analyzed by ImageJ software. In addition, Ki67 immunofluorescence staining was used to assess endometrial proliferation. Detailed information on the antibodies is listed in Tables S2 and S5 (Supporting Information).

### RNA Extraction and Reverse Transcription

The tissues and cells were treated with TRIzol reagent (Invitrogen, Carlsbad, CA) to extract total RNA. The concentration and quality of the RNA were evaluated using a NanoDrop ND‐2000 spectrophotometer (Thermo Fisher Scientific, Waltham, MA). Reverse transcription was performed using the PrimeScript RT reagent kit (TaKaRa, Shiga, Japan) with 500 ng of total RNA.

### Quantitative Real‐Time PCR

Quantitative real‐time PCR was performed using 2×SYBR Green qPCR Master Mix (Low ROX) (Bimake, USA) with an ABI Prism System (Applied Biosystems, Carlsbad, CA). All qRT‐PCRs were performed in triplicate, and the final volume of the reaction was 10 µL. Relative mRNA levels were calculated by the 2−ΔΔCt method with L19 as an internal control. Primer sequences are listed in Table S6 (Supporting Information).

### Western Blotting

The protein samples were treated with ice‐cold radioimmunoprecipitation assay (RIPA) lysis buffer (CWBIO) with a protease inhibitor cocktail (cOmplete Tablets, Mini EASYpack, Roche) and a phosphatase inhibitor (PhosSTOP EASYpack, Roche) to extract total protein from cells and tissues. The concentration of protein was determined using a Bradford assay kit (Beyotime, Shanghai, China). Subsequently, 20 µg of protein from each sample was mixed with 5 × protein loading buffer and heated at 95–100 °C for 10 min. The denatured proteins were separated by electrophoresis on a 4–12% Bis‐Tris gradient precast gel (Tanon, Shanghai, China) and transferred onto a nitrocellulose membrane. After blocking with 5% skim milk, the membranes were incubated with specific primary antibodies at 4 °C overnight, followed by incubation with horseradish peroxidase‐conjugated secondary antibodies for 1 h at room temperature. The relative intensity of the bands was detected using a chemiluminescent detection kit (Epizyme, Shanghai, China) and visualized with a G‐Box chemiluminescence image capture system (Syngene, Frederick, MD). The relative abundance of the target protein to GAPDH/ACTB was analyzed using ImageJ software and obtained as each target protein level. Primary antibodies are summarized in Table S7 (Supporting Information).

### Statistical Analysis

Statistical analysis was conducted using SPSS 26.0 and GraphPad Prism 9.0. Student's t test was used to compare two groups, while one‐way ANOVA followed by Bonferroni's tests was employed to compare three or more groups, with a significance level of *p* < 0.05 (^*^ *p* < 0.05, ^**^ *p* < 0.01, ^***^ *p* < 0.001, ns means no significance). The data are presented as the mean ± standard error of the mean (SEM). Statistical significance was determined based on data obtained from a minimum of three independent experiments.

## Conflict of Interest

The authors declare no conflict of interest.

## Author Contributions

Y.C., J.Q., and J.W. contributed equally to this work. Y.S. provided direction and supervision for the study; Y.C., J.Q., and J.W. made the experimental design and noted the performance; Y.C., J.Q., J.W., L.C., and Y.W. performed data analysis and data visualization; W.C. and X.Y. designed material for the study; Y.C., J.Q., Y.L., B.L., J.L., Y.Y., and Y.M. performed animal surgeries; L.C., Y.L., and X.C. controlled data quality; Y.S., W.C., and Y.C. wrote, reviewed, and edited the final manuscript; Y.S., Y.C., J.Q., J.W., X.C., Prof. L.G.N., and X.L. revised the manuscript.

## Supporting information

Supporting Information

## Data Availability

The data that support the findings of this study are available from the corresponding author upon reasonable request.
