# Supplementary material for: Injectable “Homing‐Like” Bioactive Short‐Fibers for Endometrial Repair and Efficient Live Births
Source: Adv Sci (Weinh). 2024 Mar 19;11(20):2306507. doi: 10.1002/advs.202306507 (PMC11132084; doi:10.1002/advs.202306507)
Supplement: Supplementary file 1 — Supporting Information [file ADVS-11-2306507-s001.pdf]

ADVANCED SCIENCE
Open Access

Supporting Information

for *Adv. Sci.*, DOI 10.1002/advs.202306507

Injectable "Homing-Like" Bioactive Short-Fibers for Endometrial Repair and Efficient Live Births

Yumeng Cao, Jia Qi, Juan Wang, Liang Chen, Yuan Wang, Yijing Long, Boyu Li, Junliang Lai, Yejie Yao, Yiwen Meng, Xiaohua Yu, Xiao-Dong Chen, Lai Guan Ng, Xinyu Li, Yao Lu, Xiaoyue Cheng, Wenguo Cui and Yun Sun**

Supporting Information for

Injectable "homing-like" Bioactive Short-Fibers for Endometrial Repair and Efficient Live Births

Yumeng Cao, Jia Qi, Juan Wang, Liang Chen, Yuan Wang, Yijing Long, Boyu Li, Junliang Lai, Yejie Yao, Yiwen Meng, Xiaohua Yu, Xiao-Dong Chen, Lai Guan Ng, Xinyu Li, Yao Lu, Xiaoyue Cheng, Wenguo Cui, and Yun Sun**

Dr. Y. Cao, Dr. J. Qi, Dr. Y. Wang, Dr. Y. Long, Dr. B. Li, Dr. J. Lai, Dr. Y. Yao, Dr. Y. Meng, Dr. X. Li, Dr. Y. Lu, Dr. X. Cheng, Prof. Y. Sun

Department of Reproductive Medicine, Ren Ji Hospital, Shanghai Jiao Tong University School of Medicine, Shanghai, China, Shanghai Key Laboratory for Assisted Reproduction and Reproductive Genetics, Shanghai 200135, China.

E-mail: syun163@163.com (Y. Sun)

Dr. J. Wang, Dr. L. Chen, Prof. W. Cui

Department of Orthopaedics, Shanghai Key Laboratory for Prevention and Treatment of Bone and Joint Diseases, Shanghai Institute of Traumatology and Orthopaedics, Ruijin Hospital, Shanghai Jiao Tong University School of Medicine, Shanghai, 200025, PR China.;

E-mail: wgcui80@hotmail.com (W. Cui)

Prof. X. Yu

Hangzhou Phil Stone Biotech Co., Ltd., Hangzhou, Zhejiang, PR China.

Prof. X. Chen

Department of Comprehensive Dentistry, University of Texas Health Science Center at San Antonio, San Antonio, TX 78229, U.S.A.; Research Service, South Texas Veterans Health Care System, Audie Murphy VA Medical Center, San Antonio, TX, 78229, U.S.A.

Prof. L. Ng

Shanghai Immune Therapy Institute, Shanghai Jiao Tong University School of Medicine affiliated Renji Hospital, 200127, Shanghai, China

Experimental Section

1. Inclusion/exclusion criteria of healthy and IUA patients

Inclusion criteria for healthy patients:

1. Females of reproductive age (20-40 years old).
2. Regular menstrual cycles.
3. Absence of known gynecological diseases or abnormalities.
4. Normal uterine anatomy confirmed by hysteroscopy.

Inclusion criteria for patients with IUA:

1. Females of reproductive age (20-40 years old).
2. Confirmed diagnosis of IUA by hysteroscopy (AFS score≥5).
3. Absence of known gynecological diseases or abnormalities except IUA.

Exclusion criteria for both healthy and IUA patients include:

1. Pregnant or current breastfeeding.

2. Presence of active pelvic infection.
3. History of malignancy.
4. History of intrauterine device use in the past 3 months.
5. History of hormones and antibiotics use in the past 3 months.
6. Polycystic ovary syndrome or other endocrine disorders.
7. Severe systemic diseases that may affect fertility or endometrial healing.
8. Inability to provide informed consent or comply with study requirements.

2. Meaning of the evaluation parameters for angiogenesis analysis

In angiogenesis analysis, different parameters are used to quantify and characterize the formation and organization of blood vessels. Here is the detailed explanation of each parameter and its significance:

Number of Segments: This parameter refers to the count of individual segments or vessel segments in the analyzed angiogenic network. It provides information about the total number of vessel segments present in the network, indicating the complexity and extent of vessel growth.

Number of Junctions: Junctions represent the points where multiple vessel segments intersect or branch out. The number of junctions indicates the level of vessel branching and connectivity in the angiogenic network. Higher values suggest a more complex and interconnected network.

Number of Branches: Branches are formed when a vessel segment splits into two or more segments at a junction. The number of branches provides insights into the level of vessel branching and the complexity of the angiogenic network. Higher values indicate a more branched and intricate network.

Number of Meshes: A mesh is formed when multiple vessel segments and junctions connect to

create a closed loop. The number of meshes reflects the presence of interconnected vessel networks and the formation of vascular loops. Higher values suggest a more interconnected and organized network.

Number of Master Segments: Master segments are the primary vessel segments that form the backbone or main branches of the angiogenic network. Counting the number of master segments helps assess the overall structure and organization of the blood vessels. Higher values indicate a more developed and organized vascular network.

Number of Master Junctions: Master junctions are the primary junctions where multiple vessel segments converge or branch out. The number of master junctions provides insights into the key points of vessel connectivity and branching in the angiogenic network. Higher values suggest a more complex and interconnected network.

Length of Total Branches: This parameter measures the cumulative length of all the vessel branches in the angiogenic network. It quantifies the overall extent of vessel growth and elongation. Higher values indicate a greater overall length of vessel branches, suggesting more extensive angiogenesis.

Length of Total Segments: Similar to the length of total branches, the length of total segments represents the cumulative length of all vessel segments in the angiogenic network. It provides a measure of the total vessel length and helps evaluate the overall vascular density. Higher values indicate a greater overall length of vessel segments, suggesting increased vessel growth and density.

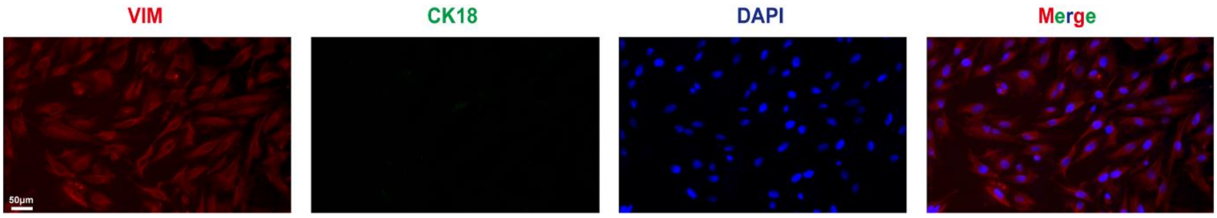

Fig. S1. Immunofluorescent staining of VIM (red) and CK18 (green) of HESCs.

Table S1. ELISA kit

Protein	Manufacturer	Catalog number
**VEGF**	Enzyme-linked Biotechnology Co., Ltd	ml256952
**EGF**	Enzyme-linked Biotechnology Co., Ltd	ml002317
**FGF**	Enzyme-linked Biotechnology Co., Ltd	ml365211
**KGF**	Enzyme-linked Biotechnology Co., Ltd	ml002348
**PDGF**	Enzyme-linked Biotechnology Co., Ltd	ml002321
**IGF-1**	Enzyme-linked Biotechnology Co., Ltd	ml002344

Table S2. Primary antibodies used for immunofluorescence

Protein	Manufacturer	Catalog number	Dilution
**Ki67**	Abcam	ab16667	1:200
**CK18**	Servicebio Technology Co., Ltd	GB11232	1:100
**VIM**	Santa Cruz Biotechnology	sc-6260	1:100
**Phalloidine**	Beyotime	C2207S	1:500

Table S3. Embryo implantation in different group

Variable	Control	Model	DEFs

**Total number of uterine horns**	24	12	12
**Number of pregnant uterine horns**	24	4	10
**Percentage of pregnant uterine horns**	100%	33.33%	83.33%
**Number of embryos implanted (mean ± SD)**	6.42±1.67	1.08±1.93	4.08±2.61

Table S4. Live birth in different group

**Variable**	**Control**	**Model**	**DEFs**
**Total number of rats**	10	10	10
**Number of giving birth rats**	10	3	9
**Percentage of giving birth rats**	100%	30%	90%
**Number of live births (mean ± SD)**	12.4±1.71	0.13±1.34	6.4±3.03

Table S5. Primary antibodies used for immunohistochemistry

**Protein**	**Manufacturer**	**Catalog number**	**Dilution**
**CD31**	R&D	AF3628	1:40
**α-SMA**	Cell Signaling Technology	19245	1:200
**VIM**	Santa Cruz Biotechnology	sc-6260	1:200
**vWF**	Servicebio Technology Co., Ltd	GB11020	1:500
**CK18**	Servicebio Technology Co., Ltd	GB11232	1:200

Table S6. Primer sequences

**Gene name (species)**	**Forward primer sequence (5’-3’)**	**Backward primer sequence (5’-3’)**
**L19**	GCCGAAGGGTACAGCC	GCAGCCGGCGCAAA

**(Homo sapiens)**	AA	
**COL4A1** **(Homo sapiens)**	TGTTGACGGCTTACCTGGAGAC	GGTAGACCAACTCCAGGCTCTC
**FOXF2** **(Homo sapiens)**	TCGCTGGAGCAGAGCTACTT	CCCATTGAAGTTGAGGACGA
**CTGF** **(Homo sapiens)**	CAGCATGGACGTTCGTCTG	AACCACGGTTTGGTCCTTGG
**α-SMA** **(Homo sapiens)**	CTATGAGGGCTATGCCTTGCC	GCTCAGCAGTAGTAACGAAGGA
L19 **(*Rattus norvegicus*)**	CTGCGTCTGCAGCCATGAGTAT	GCTTCCTGATCTGTTGACGAGAG
Col4a1 **(*Rattus norvegicus*)**	GCGAGATGTTCAAGAAGCCC	AGGAGGGAGTAGCACCATGT
Col4a2 **(*Rattus norvegicus*)**	CACCGGGGACTTTGGTGATA	AATCCAACGTCACCCTCAGC
Tgf-β1 **(*Rattus norvegicus*)**	CAGTGGCTGAACCAAGGAGA	CGTTTGGGACTGATCCCATTGA
Foxo1 **(*Rattus norvegicus*)**	GGCGGGCTGGAAGAATTCAA	ACTCTTGCCTCCCTCTGGAT
Hoxa11 **(*Rattus norvegicus*)**	CGGTGGCTCCGGTGG	ATACGGGACAGTTGCAGACG
Lif **(*Rattus norvegicus*)**	GTCTTGGCCACAGGGATTGTG	CGTTGAGTTGAGCCAGTTGAC
Igf-1	GAGCGCACCTCCAATA	CACGAACTGAAGAGCG

**(*Rattus norvegicus*)**	AAGA	TCCA
Vegfa **(*Rattus norvegicus*)**	GAAAGCCCATGAAGTG GTGA	ACACAGGACGGCTTGA AGAT
Pdgfc **(*Rattus norvegicus*)**	AGGAGCAGAACGGAGT GCAA	CCAGCACCGTATTTCTT GGG

Table S7. Primary antibodies used for Western blotting

Protein	Manufacturer	Catalog number	Dilution
**CD31**	Abcam	ab281583	1:1000
**LIF**	Abcam	Ab113262	1:1000
**CTGF**	Santa Cruz Biotechnology	sc-365970	1:500
**TGF-β1**	Santa Cruz Biotechnology	sc-130348	1:500
**HOXA11**	Santa Cruz Biotechnology	sc-393440	1:1000
**COL4A2**	Proteintech Group Inc	55131-1-AP	1:1000
**FOXO1**	Proteintech Group Inc	2880	1:1000
**ACTB**	Proteintech Group Inc	20536-1-AP	1:10000
**GAPDH**	Proteintech Group Inc	60004-1-Ig	1:10000
**COL4A1**	Cell Signaling Technology	50273	1:1000
**α-SMA**	Cell Signaling Technology	19245	1:1000
**SMAD2/3**	Cell Signaling Technology	8685	1:1000
**p-SMAD2/3 (Ser465/Ser467)**	Cell Signaling Technology	18338	1:1000
